# Natural Products Targeting Myocardial Fibrosis: Pharmacological Basis, Molecular Mechanisms and Translational Barriers

**DOI:** 10.3390/ijms27167075

**Published:** 2026-08-07

**Authors:** Tiantian Long, Junchen Ma, Weijun Hu, Qiwen Wang, Zihao Xie, Sirong Wu, Huaying Wu

**Affiliations:** Key Laboratory of Study and Discovery of Small Targeted Molecules of Hunan Province, Hunan Normal University Health Science Center, Hunan Normal University, Changsha 410013, China; ttt1_an@hunnu.edu.cn (T.L.); 2990468446@hunnu.edu.cn (J.M.); 3197778533@hunnu.edu.cn (W.H.); 202430192016@hunnu.edu.cn (Q.W.); 15272475664@163.com (Z.X.)

**Keywords:** myocardial fibrosis, natural products, cardiac remodeling, extracellular matrix, signal transduction, preclinical translation

## Abstract

Myocardial fibrosis (MF) is a hallmark of pathological cardiac remodeling, driven by extracellular matrix (ECM) accumulation, fibroblast activation, and oxidative stress. Diverse natural products—notably alkaloids, flavonoids, terpenoids, and saponins—exhibit emerging antifibrotic potential. A comprehensive literature synthesis through July 2026 across PubMed, Web of Science, and Scopus categorized these agents by phytochemical class and disease models, delineating direct antifibrotic efficacy from indirect cardioprotection. Mechanistically, these compounds target TGF-β signaling, endothelial-to-mesenchymal transition (EndMT), autophagy/mitophagy, and ECM turnover. Robust preclinical evidence correlates with integrated histopathological assessment, specifically collagen and α-SMA quantification. However, translation remains impeded by model limitations, inadequate phytochemical standardization, and a critical paucity of clinical trials. Currently, no natural product holds clinical approval for MF. Future directives necessitate rigorous botanical authentication, pharmacokinetic/pharmacodynamic profiling, and adoption of human-relevant models, such as induced pluripotent stem cell-derived cardiomyocytes, to bridge the bench-to-bedside gap.

## 1. Introduction

Cardiovascular diseases persist as the predominant contributor to global mortality, accounting for an estimated 19.8 million deaths (approximately 32% of the total) in 2022. Myocardial fibrosis (MF) represents a convergent pathological phenotype across a spectrum of cardiovascular disorders—including myocardial infarction, hypertensive heart disease, diabetic cardiomyopathy, heart failure, and cardiotoxic injury. Characterized by excessive extracellular matrix (ECM) accretion, proliferation and activation of cardiac fibroblasts and myofibroblasts, dysregulated collagen I/III stoichiometry, and progressive deterioration of ventricular compliance and electrical stability, MF serves as a critical determinant of adverse outcomes [1,2].

Conceptually, MF is a heterogeneous pathological process rather than a single, uniform lesion. While reparative fibrosis post-myocardial injury maintains structural integrity, chronic neurohumoral, metabolic, or inflammatory stress drives diffuse interstitial and perivascular fibrosis, culminating in ventricular stiffening, arrhythmogenesis, and heart failure progression [1,3]. Critically, this context-dependent duality presents a fundamental challenge in natural product research. Collagen attenuation, when observed in isolation, serves merely as a descriptive histological marker rather than a definitive index of therapeutic efficacy. A rigorous evaluation must therefore integrate disease stage, experimental fidelity, functional endpoints, and systemic safety profiles to distinguish genuine antifibrotic benefit from mere histological alteration.

Natural products represent an important source of bioactive molecules and promising lead compounds for cardiovascular drug discovery. Pharmacologically, their relevance depends not only on biological activity but also on the links among traditional or regional use, authenticated source materials, chemical composition, pharmacological plausibility, and reproducibility [4]. Indeed, many medicinal plants and formulations used to treat cardiovascular or circulation-related disorders contain compounds with anti-inflammatory, antioxidant, mitochondrial-protective, endothelial-modulating, or fibroblast-regulating effects, providing a plausible link between traditional medical practice and contemporary fibrosis research.

Despite the rapidly expanding literature on natural products for MF, the available evidence remains highly heterogeneous. Some studies directly assess MF in animal models or cultured cardiac fibroblasts, whereas others infer antifibrotic potential from cardioprotective or anti-inflammatory effects, cardiac hypertrophy models, or non-cardiac fibrosis systems. Accordingly, this review emphasizes systematic evidence mapping rather than merely cataloguing individual compounds. It integrates chemical classes, ethnomedical context, source authentication, model relevance, mechanistic pathways, and translational limitations, while avoiding overinterpretation of preclinical findings or unsupported claims of clinical efficacy in the absence of human validation.

To synthesize these complex interactions, this review adopts a structured narrative approach. We surveyed the peer-reviewed literature from PubMed, Web of Science, Scopus, and Google Scholar (through July 2026), prioritizing original studies that directly quantify antifibrotic efficacy in cardiac models or human-derived systems. While foundational concepts were informed by non-cardiac fibrosis research and general reviews, the primary focus remains on translating phytochemical discoveries into mechanistically validated cardiac insights. Notably, patent databases were excluded to ensure that our conclusions reflect rigorously validated pharmacological evidence rather than proprietary claims. By integrating ethnomedical context with molecular targets, we aim to delineate a translational roadmap for natural product drug development against MF.

## 2. Pathophysiology of MF

MF is a common pathological feature of cardiac remodeling across a wide range of cardiovascular diseases. It is characterized by excessive ECM deposition, altered collagen composition, and disruption of the spatial organization of the matrix [1]. This process occurs in diverse pathological settings, including hypertensive heart disease, myocardial infarction, diabetic cardiomyopathy, and hereditary or acquired cardiomyopathies, and is closely associated with reduced myocardial compliance, systolic and diastolic dysfunction, arrhythmias, and the progression of heart failure [5,6]. MF is not a single, homogeneous disease process but a dynamic cardiac response to diverse injurious stimuli. Its pathological significance depends on the nature of the injury, the stage of disease, and the spatial distribution of fibrosis.

Importantly, MF should be interpreted in a stage-specific context. Reparative fibrosis following acute myocardial infarction may initially stabilize the injured myocardium, whereas diffuse interstitial or perivascular fibrosis associated with chronic pressure overload, metabolic disease, aging, or cardiotoxic injury often becomes maladaptive. This distinction is essential when evaluating natural products, because reducing collagen deposition may not be beneficial at very early stages of myocardial injury.

Cardiac fibroblasts are the principal cell type responsible for maintaining myocardial ECM homeostasis. Under physiological conditions, these cells maintain tissue architecture by coordinating matrix synthesis, degradation, and remodeling [7]. In response to pathological stimuli such as pressure overload, ischemic injury, inflammation, or metabolic abnormalities, quiescent cardiac fibroblasts become activated and differentiate into myofibroblasts [2]. Myofibroblasts typically exhibit increased expression of α-smooth muscle actin (α-SMA), together with enhanced proliferative, migratory, contractile, and ECM-secretory capacities. They are the principal effector cells driving the development and persistence of MF [8]. These cells produce and secrete large amounts of matrix components, particularly type I and type III collagens, while also regulating ECM degradation, cross-linking, and spatial remodeling. Collectively, these changes result in the net accumulation of collagen-rich ECM and increased myocardial stiffness [5,9].

At the molecular level, MF is initiated by diverse etiological factors and governed by complex, interconnected networks involving neurohumoral activation, inflammatory and immune responses, oxidative stress, mechanical stress, metabolic dysregulation, and intercellular communication [1,10]. Among these factors, transforming growth factor-β1 (TGF-β1) is widely recognized as a central profibrotic regulator of cardiac remodeling [11]. TGF-β1 binds to cell-surface type II and type I TGF-β receptors, leading to the phosphorylation of Smad2/3 and their association with Smad4. The resulting complex translocates to the nucleus and induces the transcription of genes encoding collagen type I α1 chain (*COL1A1*), collagen type III α1 chain (*COL3A1*), α-smooth muscle actin (*ACTA2*), and other fibrosis-related proteins [11,12]. In addition, TGF-β1 promotes myofibroblast differentiation and ECM synthesis, modulates the balance between matrix metalloproteinases (MMPs) and tissue inhibitors of metalloproteinases (TIMPs), and engages in positive feedback with other growth factors and inflammatory mediators, thereby amplifying the fibrotic response [12,13].

Activation of the renin–angiotensin–aldosterone system (RAAS) is another major driver of MF [14]. Angiotensin II (Ang II), the principal effector of the RAAS, directly promotes cardiac fibroblast proliferation, migration, and collagen synthesis through the angiotensin II type 1 receptor (AT1R). Ang II also induces TGF-β1 expression, thereby enhancing TGF-β/Smad-dependent profibrotic signaling [15,16]. Following myocardial injury, macrophages, lymphocytes, and other immune cells infiltrate the myocardium and release proinflammatory cytokines, including tumor necrosis factor-α (TNF-α), interleukin-1β (IL-1β), and interleukin-6 (IL-6) [17,18]. These inflammatory mediators regulate fibroblast activation through the NF-κB, Janus kinase/signal transducer and activator of transcription (JAK/STAT), and mitogen-activated protein kinase (MAPK) pathways and interact with TGF-β1 signaling to accelerate fibrotic progression.

Oxidative stress is also a major contributor to MF [19]. Under pathological conditions, excessive production of reactive oxygen species (ROS) not only directly induces cardiomyocyte injury and death but also acts as a second messenger that activates profibrotic pathways, including MAPK, NF-κB, and TGF-β/Smad signaling. These processes promote fibroblast activation, collagen synthesis, and ECM deposition [20,21]. Collectively, MF is a complex pathological process driven by fibroblast phenotypic transition, dysregulated ECM turnover, mutually reinforcing inflammatory and oxidative stress responses, and the coordinated activation of multiple profibrotic pathways. A deeper understanding of these mechanisms will provide a mechanistic foundation for the development of therapeutic strategies targeting MF [3,22].

## 3. Ethnopharmacological and Pharmacological Evidence of Natural Products

In addition to conventional herbal extracts and formula-based interventions, increasing attention has focused on chemically defined natural products with potential antifibrotic activity in the myocardium. These compounds, including alkaloids, polysaccharides, flavonoids, phenolic compounds, saponins, and terpenoids, have been investigated in a range of fibrosis-related experimental models. Their reported effects involve the modulation of multiple profibrotic and inflammatory pathways, providing mechanistic support for the development of natural product-based antifibrotic strategies (Table 1).

## 4. Mechanistic Integration of Antifibrotic Natural Products

### 4.1. TGF-β/Smad Signaling Modulation

**Figure 1:**
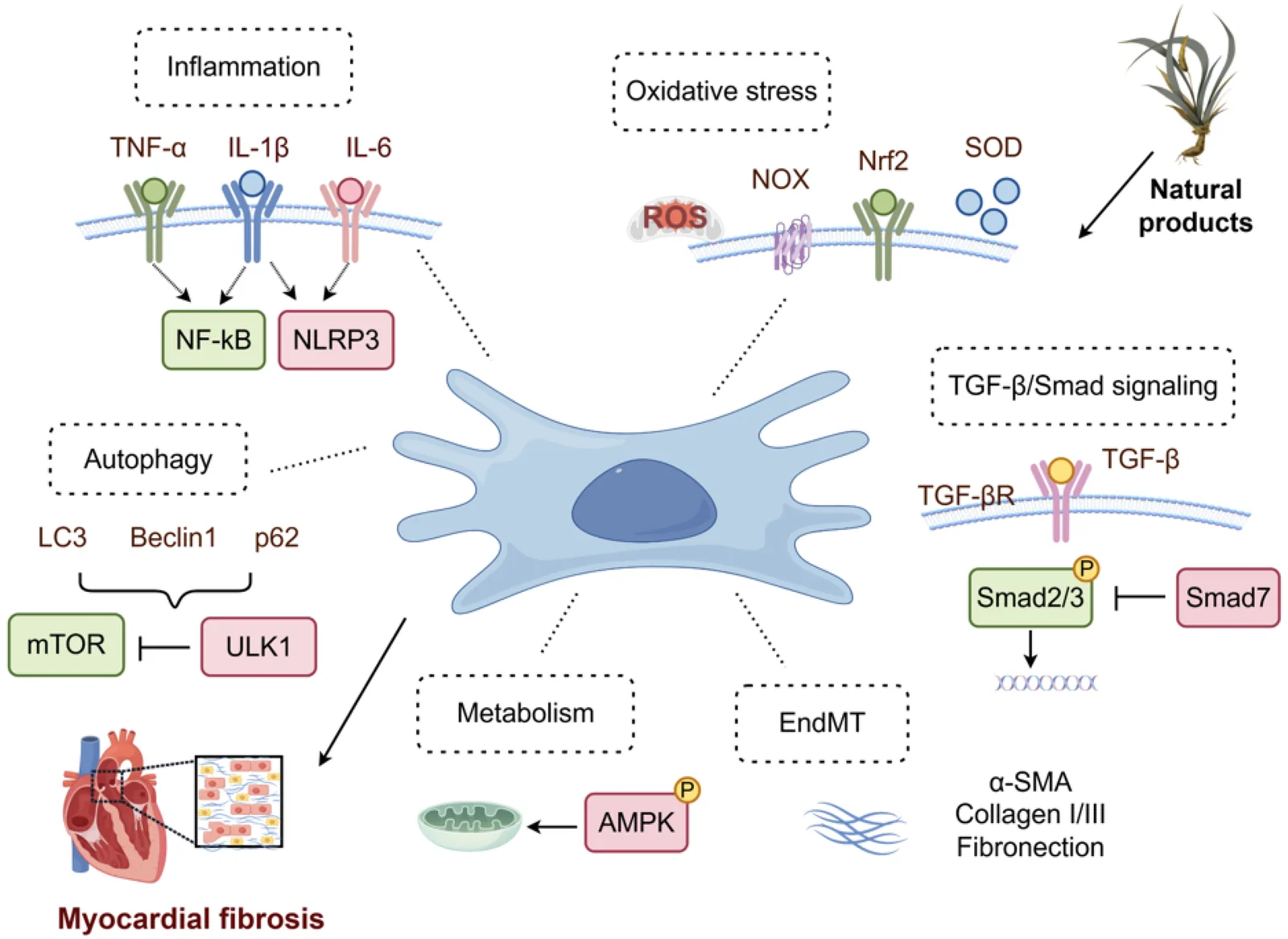
Natural products attenuate myocardial fibrosis through multitarget modulation of inflammatory responses, oxidative stress, autophagy, metabolic homeostasis, fibroblast-to-myofibroblast transition, and TGF-β/Smad signaling. These coordinated regulatory effects suppress fibroblast activation and extracellular matrix accumulation, thereby mitigating adverse cardiac remodeling.

The TGF-β/Smad pathway is a central profibrotic signaling axis in the development and progression of MF. Activation of this pathway induces Smad2/3 phosphorylation and subsequent nuclear translocation, thereby promoting cardiac fibroblast activation, myofibroblast differentiation, and excessive ECM deposition. In particular, Smad3 plays a prominent role in collagen synthesis, matrix remodeling, and scar formation, making the TGF-β/Smad pathway an important therapeutic target for natural products with antifibrotic potential (Figure 1) [49,50].

Several natural products have been reported to attenuate MF by suppressing the canonical TGF-β/Smad signaling cascade. In an isoproterenol-induced mouse model, forsythiaside B reduced the expression of TGF-β1 and Smad4 and decreased Smad3 phosphorylation, together with lower levels of α-SMA and collagen-related proteins, thereby attenuating MF [51]. Zerumbone, a sesquiterpene, suppressed TGF-β1/Smad signaling in both mouse models of myocardial infarction and complementary in vitro systems, resulting in reduced post-infarction fibrosis and improved cardiac remodeling [52]. Similarly, butein suppressed TGF-β1/Smad signaling in a mouse model of myocardial infarction and in TGF-β1-stimulated human cardiac fibroblasts, reducing the fibrotic area and improving cardiac function [53].

Other natural products exert antifibrotic effects through additional Smad-dependent mechanisms. Geniposide suppressed TGF-β1/Smad2 signaling in a pressure overload-induced model of MF, thereby reducing collagen deposition and adverse ventricular remodeling [54]. Curcumin, for which the evidence is primarily derived from in vitro studies, inhibited Smad2 and p38 MAPK activation in TGF-β1-stimulated neonatal rat cardiac fibroblasts, thereby reducing myofibroblast differentiation and type I collagen expression [39]. Ethyl ferulate has also been reported to directly target TGF-β receptor type 1 (TGFBR1), thereby attenuating after myocardial infarction [55].

Beyond suppressing receptor-mediated Smad signaling, natural products may also regulate this pathway through post-translational mechanisms. In diabetic cardiomyopathy, salvianolic acid B promoted Smad7 deubiquitination and stabilization, thereby suppressing TGF-β1 signaling and reducing MF [41]. Resveratrol attenuated fibrosis in dilated cardiomyopathy by modulating SIRT1-mediated Smad3 deacetylation [56]. Moreover, in rats with myocardial infarction, icariin reduced the levels of type I and type III collagen, MMP-2, and MMP-9 and suppressed TGF-β1 expression and Smad2/3 phosphorylation; these changes were associated with attenuated MF and ventricular remodeling [57].

However, complete blockade of TGF-β is unlikely to be an optimal long-term strategy because this cytokine also contributes to immune homeostasis and reparative tissue responses [11,58]. Accordingly, current antifibrotic efforts increasingly emphasize cell- and pathway-selective intervention, including fibroblast-restricted modulation of Smad3-dependent transcription [59] and inhibition of downstream profibrotic effectors such as IL-11 [60] or NOX4 [61], with the aim of reducing pathological matrix production while preserving physiological TGF-β functions.

### 4.2. Inflammatory Suppression

The inflammatory microenvironment is a major driver of MF initiation and progression. Following myocardial injury, macrophage–fibroblast crosstalk promotes the activation and amplification of inflammatory signaling through cytokines such as IL-1β and IL-6. This response involves the NF-κB and JAK/STAT3 pathways and the nucleotide-binding oligomerization domain-like receptor family pyrin domain-containing 3 (NLRP3) inflammasome, thereby sustaining fibroblast activation and extracellular matrix (ECM) deposition [62,63,64]. Accordingly, the anti-inflammatory and antifibrotic effects of natural products largely arise from their ability to disrupt this inflammatory cascade at multiple levels.

*Corydalis hendersonii* Hemsl. exerted complementary anti-inflammatory and antifibrotic effects by modulating NF-κB and JAK2/STAT3 signaling and reducing the expression of Ang II, MMP-2, and MMP-9 [65]. In spontaneously hypertensive rats, Icariside II reduced the expression of NF-κB p65, IL-1β, and TNF-α, accompanied by attenuated MF and improved cardiac function [66]. In an isoproterenol-induced model, Stevioside suppressed the myocardial NF-κB/TGF-β1/Smad signaling axis and reduced the expression of type I and type III collagen and α-SMA, thereby attenuating fibrotic progression [47].

Recent evidence suggests that celastrol attenuates post-infarction fibrosis and improves cardiac function, potentially through inhibition of NLRP3 inflammasome activation and reduction in IL-1β and IL-18 levels [45]. Phloretin [67] and muscone [68] have also been reported to attenuate inflammation, fibrosis, and cardiac remodeling in models of myocardial infarction by suppressing NLRP3/caspase-1/IL-1β-mediated inflammatory responses.

**Figure 2:**
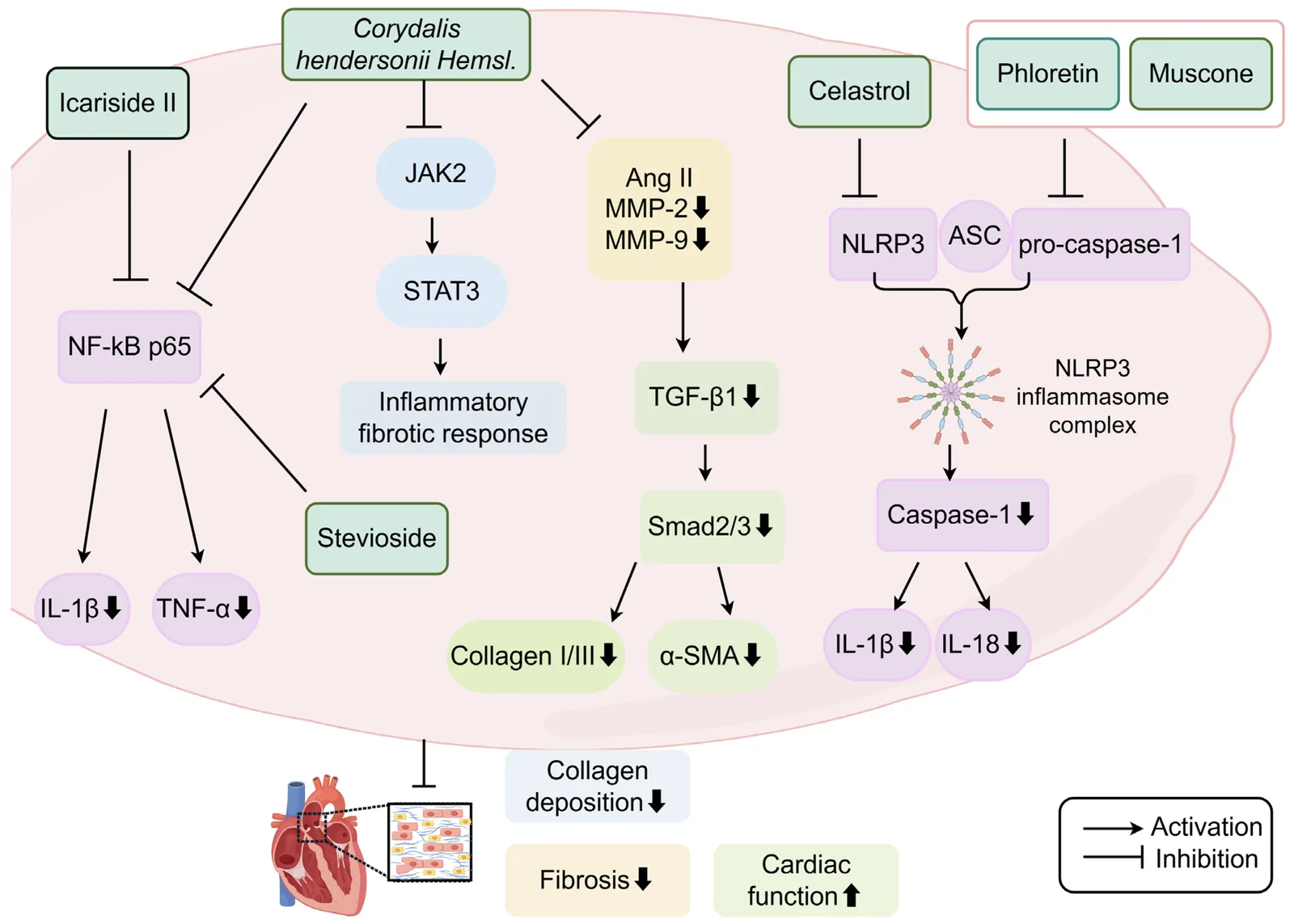
This figure illustrates the anti-inflammatory pathways. Icariside II, *Corydalis hendersonii* Hemsl., stevioside, celastrol, phloretin, and muscone are mapped to key inflammatory and profibrotic signaling axes, including NF-κB, JAK2/STAT3, TGF-β/Smad, and NLRP3/caspase-1 pathways. By suppressing cytokine release and inflammasome activation, these natural products may contribute to reduced collagen deposition and improved cardiac function.

Overall, the effects of natural products are not mediated by any single inflammatory factor. Rather, they reshape the proinflammatory microenvironment by concurrently modulating NF-κB, JAK/STAT3, and NLRP3 signaling, together with inflammatory cell–fibroblast communication, thereby producing coordinated anti-inflammatory and antifibrotic effects (Figure 2).

### 4.3. Oxidative Stress Inhibition

**Figure 3:**
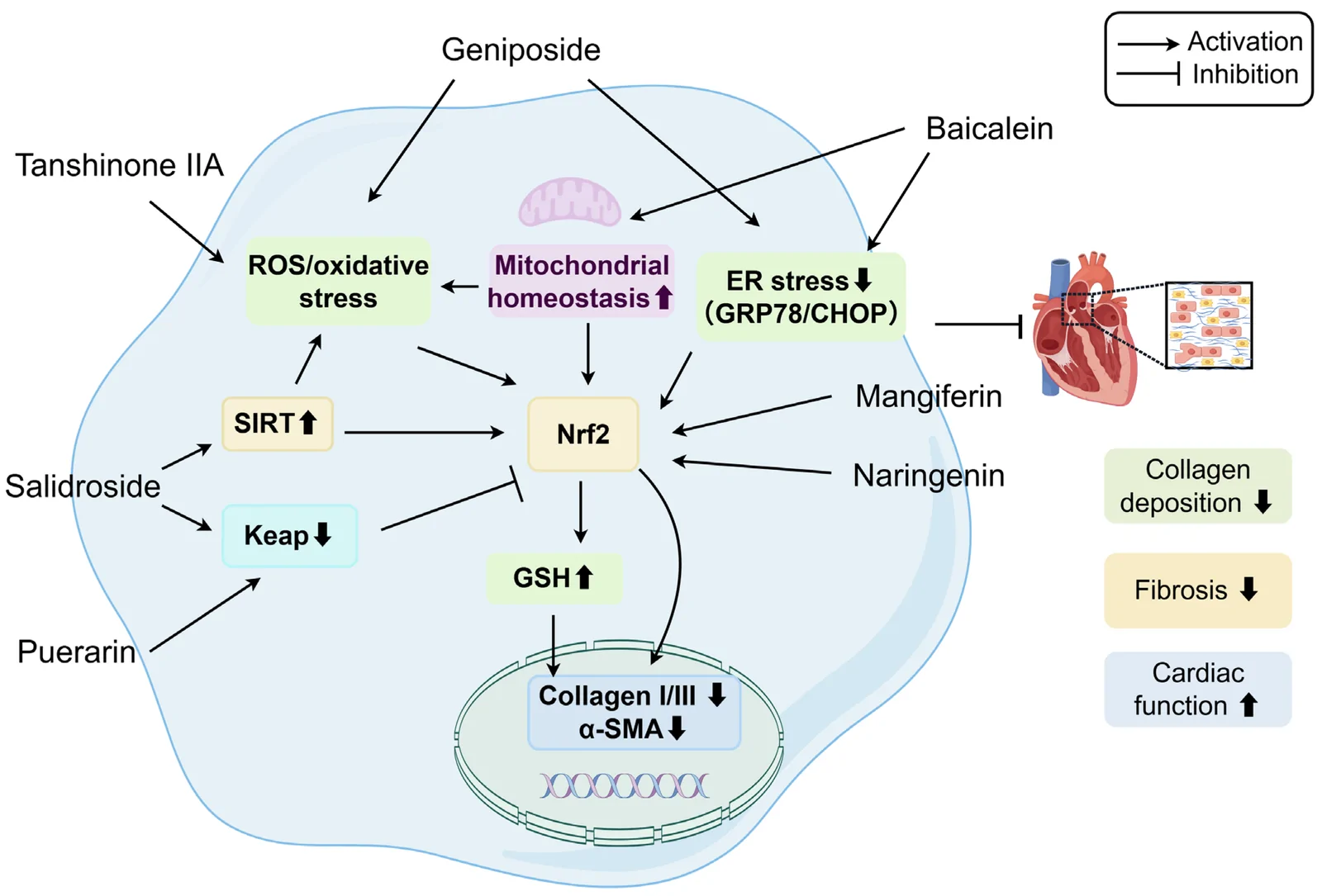
This figure illustrates the antioxidant mechanisms by which natural products modulate myocardial fibrosis. Tanshinone IIA, geniposide, salidroside, puerarin, mangiferin, naringenin, and baicalein are mapped to key redox-related processes, including ROS suppression, Keap1/Nrf2 pathway activation, mitochondrial protection, endoplasmic reticulum stress inhibition, and GSH restoration. Collectively, these effects contribute to decreased collagen I/III and α-SMA expression, thereby attenuating myocardial fibrotic remodeling.

Oxidative stress is a major pathological driver of MF. Following myocardial injury, mitochondrial dysfunction, nicotinamide adenine dinucleotide phosphate (NADPH) oxidase activation, and metabolic disturbances can lead to excessive accumulation of reactive oxygen species (ROS), resulting in lipid peroxidation and oxidative damage to proteins and deoxyribonucleic acid (DNA). Excessive ROS accumulation also activates the NF-κB and TGF-β/Smad signaling pathways, thereby promoting cardiac fibroblast activation, myofibroblast differentiation, and collagen deposition. Collectively, oxidative stress is a key process that drives adverse myocardial remodeling and fibrotic progression (Figure 3) [69,70].

Importantly, mitochondrial ROS and NADPH oxidase-derived ROS should not be regarded as interchangeable. In cardiac fibroblasts, NOX4 mediates TGF-β-induced ROS production, Smad2/3 activation and myofibroblast differentiation [61], whereas mitochondrial complex III-derived ROS can amplify TGF-β signaling and NOX4 expression, although this evidence was obtained mainly in lung fibroblasts [71]. In cardiac pressure-overload models, the mitochondria-targeted antioxidant coenzyme Q (MitoQ) reduced mitochondrial ROS, NOX4/Smad2 signaling, fibrosis and ventricular dysfunction [72]. Therefore, natural-product studies should distinguish mitochondrial and NOX-derived ROS using source-specific probes or genetic interventions rather than relying only on total ROS, malondialdehyde (MDA), SOD or GSH-Px measurements [73].

Available evidence suggests that natural products may attenuate MF by scavenging ROS, enhancing the activities of superoxide dismutase (SOD) and glutathione peroxidase (GSH-Px), restoring mitochondrial homeostasis, or activating the Nrf2/heme oxygenase 1 (HO-1) antioxidant pathway. In a model of post-myocardial infarction cardiac remodeling, andrographolide enhanced Nrf2 signaling, reduced oxidative stress, and attenuated MF [74]. In an isoproterenol-induced model of MF, geniposide improved cardiac function and reduced collagen deposition by concurrently suppressing oxidative stress and endoplasmic reticulum stress [75]. Tanshinone IIA reduced oxidative stress and attenuated fibrosis in a post-myocardial infarction model of heart failure [76]. Baicalein attenuated isoproterenol-induced fibrosis and adverse cardiac remodeling by restoring mitochondrial homeostasis, suppressing GRP78/CHOP-associated endoplasmic reticulum stress, and reducing oxidative injury [77].

In addition, puerarin attenuated cardiac fibrosis by downregulating Keap1, and enhancing Nrf2 expression and nuclear translocation [37]. Salidroside attenuated fibrosis in an Ang II-induced model of MF by activating SIRT1/Nrf2 signaling and suppressing intracellular ROS accumulation [78]. Mangiferin inhibited the differentiation of cardiac fibroblasts into myofibroblasts by promoting Keap1 degradation, stabilizing Nrf2, and increasing GSH synthesis, thereby alleviating pressure overload-induced MF [79]. In a streptozotocin (STZ)-induced model of diabetic cardiomyopathy, naringenin partially attenuated diabetes-related cardiac injury by activating Nrf2 signaling, with a concomitant reduction in MF [80].

Overall, natural products may attenuate MF by restoring redox homeostasis and disrupting inflammatory and profibrotic signaling cascades driven by oxidative stress. These effects involve multiple mechanisms, including activation of Nrf2-dependent antioxidant responses, restoration of mitochondrial function, inhibition of endoplasmic reticulum stress, and suppression of ROS-mediated fibroblast activation and ECM deposition.

### 4.4. EndMT Inhibition

Endothelial-to-mesenchymal transition (EndMT) has been proposed as a potential cellular source of myofibroblast-like cells in MF. In response to pathological stimuli such as myocardial injury, pressure overload, ischemia, or hypoxia, a subset of endothelial cells progressively loses endothelial markers, including cluster of differentiation 31 (CD31), VE-cadherin, and endothelial nitric oxide synthase (eNOS), while acquiring mesenchymal markers such as α-SMA, vimentin, and fibroblast-specific protein 1 (FSP1). This phenotypic transition is accompanied by enhanced migratory capacity and increased extracellular matrix (ECM) production, thereby contributing to the accumulation of myofibroblast-like cells and adverse myocardial remodeling [81,82].

Nevertheless, the quantitative contribution of EndMT to the activated fibroblast pool remains debated. An early study combining *Tie1*-Cre lineage tracing with fibroblast-marker analysis suggested a substantial endothelial contribution to cardiac fibrosis [83]. However, subsequent inducible lineage-tracing studies showed that fibroblast accumulation after pressure overload results predominantly from the activation and proliferation of pre-existing resident fibroblast populations, with no appreciable conversion of adult endothelial cells [84], and that periostin-expressing myofibroblasts after myocardial infarction arise mainly from *Tcf21*-lineage resident fibroblasts, with little endothelial contribution [85]. Apparent co-expression of endothelial and mesenchymal markers may instead reflect transient endothelial plasticity after injury rather than complete conversion into matrix-producing fibroblasts [86]. Developmentally derived fibroblasts of endothelial ancestry and limitations in marker or Cre-line specificity may further complicate interpretation. Thus, EndMT may contribute to endothelial dysfunction and profibrotic intercellular signaling, but in many adult cardiac fibrosis models it probably accounts for only a minor proportion of activated fibroblasts; its estimated magnitude remains dependent on the disease model, stage, markers, and lineage-tracing strategy.

Available evidence suggests that natural products may suppress EndMT by modulating signaling networks involving TGF-β/Smad, peroxisome proliferator-activated receptor gamma (PPARγ), sirtuin 1 (SIRT1), and Notch1, thereby attenuating MF. In a pressure overload-induced model of MF, puerarin suppressed EndMT and reduced collagen deposition by upregulating PPARγ and inhibiting TGF-β1/Smad2 signaling. Apigenin attenuated MF and suppressed EndMT by reducing the expression of GTP-binding protein 4 (GTPBP4) [87]. Ginsenoside Rb1 also suppressed M1 macrophage-induced, insulin-like growth factor-binding protein 2 (IGFBP2)-mediated EndMT, thereby attenuating MF in mice with chronic heart failure [88]. Diosgenin also reversed EndMT in an arsenic trioxide-induced model of MF by regulating the glucocorticoid receptor (GR)/IL-6 axis, accompanied by improved cardiac function and reduced fibrosis [89].

**Figure 4:**
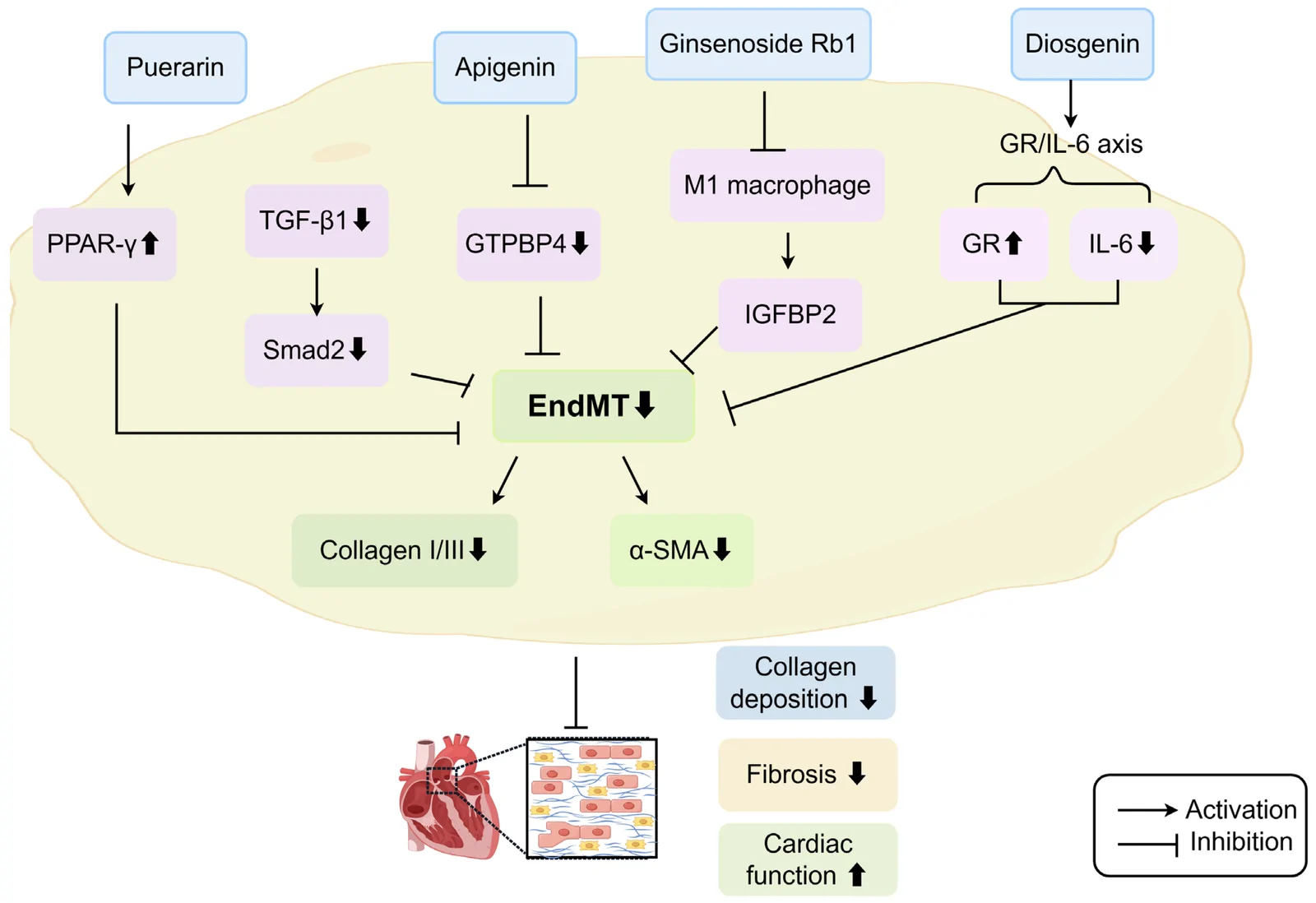
This figure summarizes the regulatory mechanisms by which natural products inhibit endothelial-to-mesenchymal transition-associated profibrotic remodeling. Puerarin, apigenin, ginsenoside Rb1, and diosgenin are mapped to key signaling axes, including PPARγ/TGF-β/Smad2, GTPBP4, macrophage-derived IGFBP2, and GR/IL-6 pathways. Collectively, these effects contribute to the maintenance of endothelial identity and the suppression of collagen deposition, thereby limiting myocardial fibrotic progression.

Overall, natural products may modulate endothelial plasticity and attenuate EndMT-associated profibrotic signaling through TGF-β/Smad, PPARγ, Nrf2, and SIRT1/Notch1 pathways. However, these effects should be interpreted as a context-dependent auxiliary mechanism rather than evidence that EndMT is a dominant source of cardiac myofibroblasts. Rigorous validation requires inducible lineage tracing combined with fibroblast-specific functional markers and matrix-production assays (Figure 4).

### 4.5. Cardiac Fibroblast Activation Inhibition

**Figure 5:**
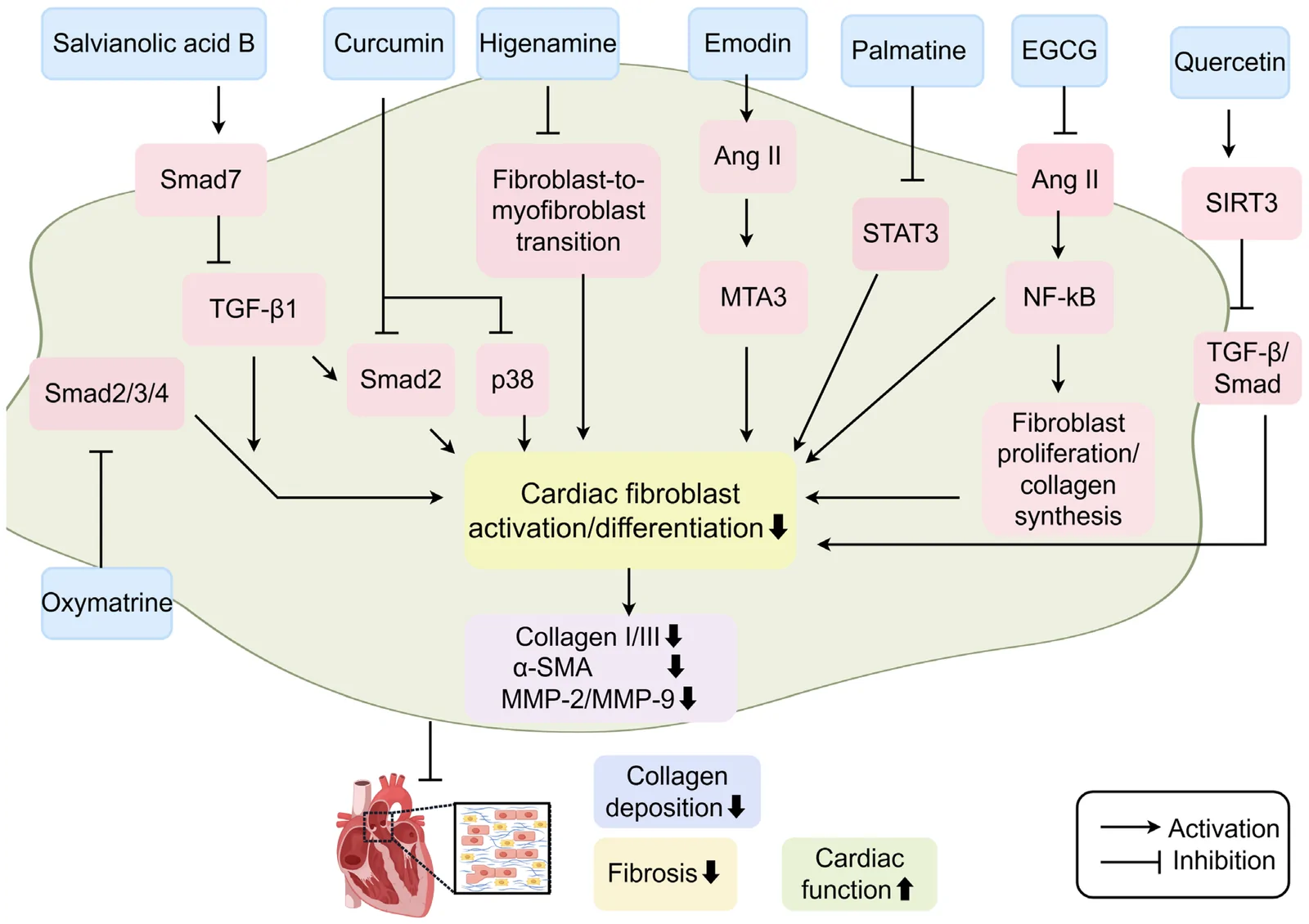
This figure illustrates the regulatory mechanisms by which natural products modulate cardiac fibroblast activation and differentiation. Salvianolic acid B, curcumin, higenamine, oxymatrine, emodin, palmatine, EGCG, and quercetin converge on key profibrotic signaling axes, including Smad, p38, STAT3, NF-κB, MTA3, and SIRT3/TGF-β/Smad pathways. Collectively, these effects contribute to the inhibition of fibroblast proliferation, myofibroblast transdifferentiation, collagen synthesis, and extracellular matrix accumulation, thereby attenuating myocardial fibrotic remodeling.

Cardiac fibroblast activation is a pivotal cellular event in the initiation and progression of MF and a major determinant of the extent of pathological cardiac remodeling [1,2,5]. Following myocardial injury, quiescent cardiac fibroblasts proliferate, migrate, and differentiate into myofibroblasts in response to profibrotic stimuli including TGF-β1, Ang II, aldosterone, high glucose, and inflammatory mediators. This phenotypic transition is characterized by increased expression of α-SMA, type I and type III collagen, fibronectin, and connective tissue growth factor (CTGF), ultimately resulting in excessive ECM deposition, reduced myocardial compliance, and adverse cardiac remodeling (Figure 5) [2,90,91].

Accumulating evidence suggests that natural products may attenuate MF by suppressing aberrant cardiac fibroblast activation and differentiation into myofibroblasts. Several compounds act predominantly by modulating the TGF-β/Smad axis. Oxymatrine inhibits aldosterone-induced cardiac fibroblast proliferation and differentiation, reduces the secretion of type I and type III collagen, and downregulates Smad2, Smad3, and Smad4 expression [92]. Salvianolic acid B suppresses high glucose-induced cardiac fibroblast proliferation, migration, differentiation into myofibroblasts, and collagen secretion, partly by promoting Smad7 deubiquitination and stabilization and thereby suppressing TGF-β1 signaling [41]. Curcumin suppresses TGF-β1-induced cardiac fibroblast differentiation by inhibiting Smad2 and p38 MAPK signaling [39]. Similarly, higenamine attenuates pressure overload-induced MF by inhibiting TGF-β1/Smad-mediated differentiation of cardiac fibroblasts into myofibroblasts [93].

Other natural products modulate additional profibrotic signaling networks. Emodin attenuates Ang II-induced cardiac fibroblast activation by upregulating metastasis-associated protein 3 (MTA3) [94], whereas palmatine suppresses fibroblast activation and fibrosis, at least in part, through inhibition of signal transducer and activator of transcription 3 (STAT3) signaling [27]. Epigallocatechin-3-gallate (EGCG) reduces Ang II-induced cardiac fibroblast proliferation, collagen synthesis, and fibronectin and CTGF expression, effects associated with the suppression of NF-κB activation [95]. Quercetin attenuates transverse aortic constriction (TAC)-induced cardiac fibrosis and inhibits cardiac fibroblast-to-myofibroblast differentiation in vitro through the SIRT3/TGF-β/Smad3 pathway [96].

Collectively, natural products may suppress pathological cardiac fibroblast activation, reduce ECM accumulation, and limit adverse cardiac remodeling by modulating the TGF-β/Smad, STAT3, NF-κB, MAPK, and SIRT-related pathways and their interconnected regulatory networks.

### 4.6. Autophagy Modulation

In MF, autophagy is not an isolated regulatory node but a dynamic process closely linked to cardiomyocyte injury, mitochondrial dysfunction, and fibroblast activation. The core molecular machinery includes autophagy initiation by the unc-51-like autophagy-activating kinase 1 (ULK1) complex [97], membrane nucleation mediated by the Beclin 1-vacuolar protein sorting 34 (Vps34) complex [98], microtubule-associated protein 1 light chain 3 (LC3) lipidation, and sequestosome 1 (p62-dependent cargo recognition and delivery). Upstream regulation is primarily mediated by the AMP-activated protein kinase–mechanistic target of rapamycin–ULK1 (AMPK/mTOR/ULK1) axis and the PTEN-induced kinase 1 (PINK1)/Parkin mitophagy pathway [99]. Notably, under certain pathological conditions, including heart failure, dysregulated or excessive autophagy may itself contribute to cardiac fibroblast activation.

An important methodological consideration is that an increased LC3-II/LC3-I ratio or elevated Beclin 1 expression does not necessarily indicate enhanced autophagic flux. More robust assessment requires lysosomal inhibition assays, tandem fluorescent LC3 reporters, measurements of p62 turnover, electron microscopy, or genetic modulation of the autophagy machinery. Moreover, autophagy in cardiomyocytes, cardiac fibroblasts, and macrophages, as well as mitophagy, may have distinct or even opposing effects depending on the disease model and stage.

Against this background, several natural products have been reported to attenuate MF by restoring defective autophagic activity, particularly autophagy–lysosomal flux. In rats with diabetic cardiomyopathy, luteolin relieved c-Jun N-terminal kinase (JNK)/c-Jun/microRNA-221 (miR-221)-mediated suppression of autophagy, as indicated by decreased p62 expression, an increased LC3-II/LC3-I ratio, and increased autophagosome formation, these changes were accompanied by improved cardiac function and reduced fibrosis [100]. In an isoproterenol-induced model of MF and atrial fibrillation-associated remodeling, quercetin promoted autophagy by inhibiting miR-223-3p and upregulating forkhead box O3 (FOXO3) and autophagy-related proteins, thereby suppressing collagen deposition and myocardial remodeling [101]. In a pressure overload-induced model of heart failure, puerarin restored impaired autophagy–lysosomal function, an effect associated with reduced myocardial hypertrophy and fibrosis [102].

Chikusetsu saponin IVa restored autophagic activity by activating AMPK, inhibiting mTOR, and reducing inhibitory phosphorylation of ULK1 at Ser757, thereby attenuating isoproterenol-induced MF in mice [43]. These findings identify the AMPK/mTOR/ULK1 axis as one of the better-characterized autophagy-related pathways through which natural products exert antifibrotic effects in MF.

In a post-myocardial infarction model, crocetin enhanced autophagic flux, as indicated by increased LC3-II levels and autolysosome formation, promoted autophagosome–lysosome maturation, and inhibited fibroblast-to-myofibroblast differentiation, thereby reducing post-infarction fibrosis [103]. Chlorogenic acid upregulated PINK1 and Parkin expression and enhanced autophagic flux. In the presence of lysosomal inhibitors, the further accumulation of Parkin, p62, and LC3-II provided additional evidence of enhanced autophagic flux [104].

**Figure 6:**
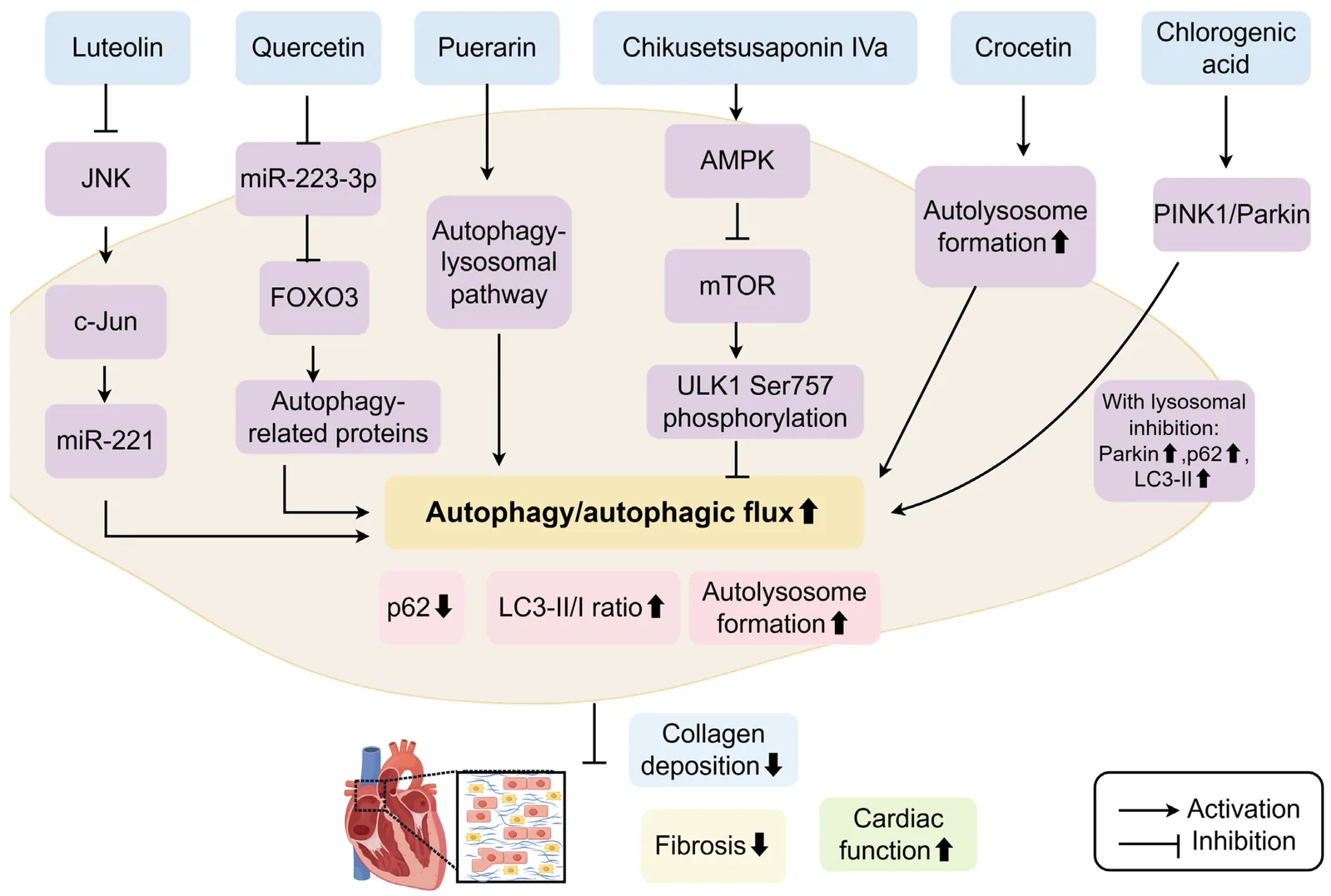
This figure illustrates the autophagy-associated regulatory mechanisms by which natural products attenuate myocardial fibrosis. Luteolin, quercetin, puerarin, Chikusetsusaponin IVa, crocetin, and chlorogenic acid are mapped to key autophagy-related signaling pathways and processes, including JNK/c-Jun/miR-221, miR-223-3p/FOXO3, autophagy–lysosomal pathway regulation, AMPK/mTOR/ULK1 signaling, autolysosome formation, and PINK1/Parkin-mediated mitophagy. Collectively, these effects underscore the restoration of autophagic flux, rather than a simple increase in LC3 expression, as the central mechanistic interpretation of autophagy-mediated antifibrotic activity.

Collectively, the available evidence suggests that the autophagy-related mechanisms by which natural products attenuate MF should not be interpreted simply on the basis of increased LC3 or Beclin 1 expression. Rather, their central effects may involve the restoration of impaired autophagy–lysosomal flux through the AMPK/mTOR/ULK1 axis and PINK1/Parkin-mediated mitophagy. By improving mitochondrial quality control, these compounds may reduce ROS production and inflammatory amplification, thereby limiting cardiac fibroblast activation, myofibroblast differentiation, and collagen deposition (Figure 6).

### 4.7. Other Regulatory Mechanisms

Recent evidence suggests that sustained endoplasmic reticulum stress, dysregulated ECM remodeling, aberrant activator protein-1/interleukin-11 (AP-1/IL-11) signaling, excessive activation of downstream RAAS effectors, and abnormal upregulation of Wnt/β-catenin signaling can all promote collagen deposition and adverse cardiac remodeling. Accordingly, beyond the major mechanisms discussed above, modulation of these processes may represent an important complementary means by which natural products exert antifibrotic effects in MF [105,106].

With respect to endoplasmic reticulum stress and apoptosis, baicalein has been shown to improve cardiac function and attenuate MF in an isoproterenol-induced model of heart failure. These effects were associated with suppression of GRP78/CHOP-associated endoplasmic reticulum stress, restoration of mitochondrial function, and reduced cardiomyocyte apoptosis [77]. Earlier studies also showed that baicalein reduced the expression and activity of MMP-2 and MMP-9, accompanied by improvements in MF and ventricular remodeling, suggesting an additional role in the regulation of ECM turnover [107].

In an isoproterenol-induced model of heart failure and in primary cardiac fibroblasts, sodium houttuyfonate significantly reduced the expression of collagen I, α-SMA, MMP-2, and TIMP-2 in isoproterenol-induced heart failure mice and primary cardiac fibroblasts, thereby attenuating MF progression [108].

In an isoproterenol-induced model of MF, 5-demethylnobiletin reduced collagen deposition and cardiomyocyte apoptosis. Its mechanism was associated with inhibition of Wnt/β-catenin signaling and the simultaneous activation of the protective SIRT1/FOXO3 pathway [106]. Because Wnt/β-catenin signaling contributes to the maintenance of the activated fibroblast phenotype and ECM deposition during cardiac fibrosis, flavonoids and related polymethoxyflavones warrant further investigation as potential antifibrotic candidates.

With respect to AP-1/IL-11 signaling, lutein has been reported to inhibit Ang II-induced cardiac remodeling. RNA sequencing (RNA-seq) analysis showed that IL-11 was a key mediator significantly upregulated in response to Ang II, whereas lutein downregulated IL-11 expression by inhibiting AP-1 activity, accompanied by lower expression of fibrosis-related genes and attenuated cardiac remodeling [109]. This finding is particularly relevant because IL-11 has recently emerged as an important downstream effector of cardiac fibrogenesis.

In an Ang II-induced model of cardiac fibrosis, ginsenoside Rg3 upregulated glucagon-like peptide-1 receptor (GLP-1R) and inhibited activation of the Ras homolog family member A/Rho-associated coiled-coil-containing protein kinase (RhoA/ROCK) pathway. These changes were accompanied by reduced expression of α-SMA, type I and type III collagen, MMP-2, and MMP-9, together with attenuated MF [110]. These findings suggest that natural products may attenuate not only classical Ang II-driven injury but also fibrotic remodeling through modulation of metabolic–cardiovascular crosstalk pathways, including GLP-1R signaling.

### 4.8. Cross-Study Convergence, Mechanistic Confidence and Translational Prioritization

Synthesizing evidence across phytochemical classes reveals that reported antifibrotic mechanisms coalesce around a finite set of interrelated signaling hubs rather than disparate molecular targets. The TGF-β/Smad axis constitutes the preeminent hub, consistently modulated by alkaloids, flavonoids, phenolics, saponins, and terpenoids across diverse pathological contexts—including myocardial infarction, pressure overload, and diabetic cardiomyopathy. Convergent with this is a second tier comprising NF-κB/NLRP3- and JAK/STAT3-mediated inflammatory cascades, alongside the Keap1/Nrf2/ROS-mitochondrial network, which synergistically drive fibroblast activation and extracellular matrix (ECM) accrual [27,65,111]. Accordingly, fibroblast-to-myofibroblast transition and dysregulated ECM turnover represent the principal downstream phenotypic hub shared by these pathways [59,112].

Current evidence lends the highest confidence to three mechanistic pillars: TGF-β/Smad suppression, Nrf2-dependent restoration of redox homeostasis, and inhibition of NF-κB/NLRP3 inflammasome signaling [79,113,114]. These pillars are buttressed by multiple chemically distinct compounds, validated across heterogeneous disease models, and corroborated by direct fibrotic endpoints—ranging from histopathological collagen quantification to improvements in cardiac function. In contrast, the evidence base for autophagy/mitophagy remains intermediate; while studies assessing autophagic flux support AMPK/mTOR/ULK1 and PINK1/Parkin axes [104,115], conclusions predicated solely on surrogate markers (e.g., LC3-II or Beclin-1 abundance) lack the resolution to define mechanistic causality.

By contrast, natural-product-associated EndMT mechanisms [116], gut microbiota-derived 12-hydroxyeicosapentaenoic acid (12-HEPE) signaling [30], GTPBP4-dependent ribosome biogenesis [87], macrophage-derived *IGFBP2* signaling [88], AP-1/IL-11 signaling [109], and GLP-1R/RhoA/ROCK signaling [110] currently represent emerging rather than established mechanistic explanations. Support for these compound–pathway linkages are largely confined to single or small-scale studies, lacking independent replication across laboratories. While individual reports employ sophisticated perturbations, the overarching evidence often falls short due to the absence of endothelial lineage tracing, cell-type-specific genetic manipulations in vivo, or direct compound–target engagement assays.

Despite extensive preclinical activity, no natural product in this review has achieved clinical validation for MF [70]. Puerarin emerges as the leading candidate, exhibiting a robust evidence chain across multiple cardiac fibrosis models, with mechanistic convergence on TGF-β/Smad, Nrf2, EndMT, and autophagy [36,117]. Salvianolic acid B and tanshinone IIA form a second high-priority group, supported by well-defined chemistry and direct cardiac-model evidence [41,76,118]. While berberine and quercetin show promise, their development is hindered by variable bioavailability and insufficient MF-specific replication [96,119]. Conversely, curcumin and celastrol, despite potent antifibrotic mechanisms, face unfavorable developability profiles, including poor solubility, metabolic instability, and narrow therapeutic windows [120].

Crucially, in the absence of head-to-head comparisons, “potency” should not be construed as a strict pharmacological ranking. Instead, based on evidence breadth, mechanistic depth, and translational feasibility, we prioritize five lead candidates: puerarin, salvianolic acid B, tanshinone IIA, berberine, and quercetin.

## 5. Translational Barriers, Safety Risks and Quality Control Standardization

The translational gap remains the core bottleneck restricting the clinical development of natural product-derived antifibrotic candidates. Although many compounds reduce fibrosis-related biomarkers in rodent models or cultured cardiac cells, only a minority have been evaluated through an integrated pharmacokinetic/pharmacodynamic framework that includes oral bioavailability, metabolism, tissue distribution, target engagement, repeated-dose exposure, long-term safety and human-relevant therapeutic endpoints [121]. Oral dose should not be treated as a surrogate for myocardial exposure [122]. For example, resveratrol is absorbed but undergoes rapid intestinal and hepatic sulfation and glucuronidation, leaving very low circulating concentrations of unchanged compound [123]. Accordingly, future studies should quantify the parent compound and major active or toxic metabolites, area under the concentration–time curve (AUC), half-life, protein binding, dose proportionality and accumulation, and establish exposure–response relationships in plasma and, where feasible, cardiac tissue.

Mechanistic translation also requires evidence that the candidate engages the proposed target at pharmacologically achievable concentrations. Changes in phosphorylated Smad proteins, Nrf2, NF-κB or autophagy markers demonstrate pathway association but do not establish direct target binding or cellular target occupancy [124]. Compound progression should therefore incorporate orthogonal approaches such as cellular thermal shift assays, thermal proteome profiling, chemoproteomics, drug-affinity responsive target stability, loss-of-function experiments and pharmacological rescue [125,126], ideally at unbound concentrations matched to in vivo exposure. For multicomponent extracts, target engagement should be linked either to defined active constituents or to a reproducible chemical fingerprint rather than being inferred from network-pharmacology predictions alone.

Formulation development is consequently not a secondary technical issue but a central determinant of translational feasibility. Potential strategies include micellar and cyclodextrin systems, phospholipid complexes, lipid or polymeric nanoparticles, prodrugs and controlled-release preparations. In a randomized human crossover study, micellar and γ-cyclodextrin formulations markedly increased curcumin exposure relative to native curcumin, although circulating curcumin remained predominantly conjugated rather than free [127]. Formulations should therefore be compared using parent and metabolite-specific PK, myocardial delivery, safety, manufacturability, stability and batch reproducibility, rather than total plasma exposure alone. Nanocarrier-based approaches additionally require evaluation of biodistribution, carrier-related toxicity, immunogenicity and long-term retention [128].

Any clinical application would most plausibly be as an adjunct to, rather than a replacement for, guideline-directed medical therapy (GDMT). Patients with heart failure commonly receive combinations of renin–angiotensin system inhibitors or angiotensin receptor–neprilysin inhibitors, evidence-based β-blockers, mineralocorticoid receptor antagonists, sodium-glucose cotransporter-2 inhibitors, diuretics and, according to comorbidity, antiplatelet, anticoagulant or lipid-lowering therapy [129]. Natural products may alter cytochrome P450 (CYP) enzymes, UDP-glucuronosyltransferases, P-glycoprotein and uptake transporters, or may produce additive effects on blood pressure, heart rate, potassium, renal function and bleeding risk; clinically relevant interactions have been reported for cardiovascular herbal medicines, including Danshen and ginseng-containing products [130,131,132]. Future trials should therefore require stable background GDMT, prospectively record concomitant medication, perform interaction-focused PK and safety monitoring, and distinguish the incremental effect of the candidate from the effect of GDMT initiation or optimization.

Clinical experience indicates that improved formulation or biomarker modulation does not necessarily translate into clinical benefit. In a randomized trial of a high-absorption curcumin formulation in patients with early hypertensive heart disease, curcumin reduced the increase in plasma B-type natriuretic peptide but did not improve the primary endpoint of left ventricular diastolic function [133]. Such discordant findings may reflect insufficient myocardial exposure, short treatment duration, heterogeneous disease stages, limited sample size, insensitive endpoints, or background therapy. Future trials should therefore establish human PK/PD and target engagement, enrich patients using imaging or validated fibrosis biomarkers, and integrate structural, functional, and clinical outcomes [121,134]. Regulatory requirements also differ among purified compounds, standardized extracts, and multicomponent botanical products. Traditional-use registration pathways should be distinguished from conventional marketing authorization because they may rely partly on longstanding use while still requiring pharmaceutical quality control.

Rigorous quality control is an essential prerequisite for reliable preclinical research and subsequent clinical translation. For purified single compounds, comprehensive data on chemical purity, structural stability, metabolite profiles, and in vivo exposure are essential for ensuring reproducible pharmacological evaluation. By contrast, crude extracts, polysaccharides and complex traditional formulas require more extensive and standardized quality documentation, including authentication of the source materials, complete botanical or organismal identification, preservation of voucher specimens or equivalent traceability records, standardized preparation protocols, batch information, plant-to-extract ratios, characteristic chemical fingerprints, quantitative analysis of marker compounds, molecular weight distributions of polysaccharides, and systematic screening for exogenous contaminants.

A major limitation of current preclinical antifibrotic research is the widespread misuse and oversimplification of disease models, which substantially reduce the translational relevance of findings on natural products. Most existing studies rely on a single acute-injury model and fail to distinguish between the two major forms of MF: adaptive reparative fibrosis and pathological maladaptive fibrosis. This distinction is critical because it may determine whether an intervention is beneficial or detrimental. To address this limitation, future preclinical studies should use disease models that faithfully recapitulate the heterogeneous, clinically relevant phenotypes of human MF, including post-infarction reparative remodeling, chronic maladaptive interstitial and perivascular fibrosis induced by sustained pressure overload, metabolic fibrotic remodeling in diabetic cardiomyopathy, and progressive cardiotoxic fibrosis induced by anthracycline chemotherapy.

Rigorous multiparametric evaluation frameworks are essential to avoid incomplete or superficial assessments of efficacy. Fibrotic phenotypes should be comprehensively evaluated using complementary approaches, including histological staining to characterize the morphology and distribution of fibrotic lesions, biochemical quantification of total collagen and hydroxyproline levels, molecular assessment of fibrotic markers including collagen I/III ratio and α-SMA expression, and systematic profiling of the balance between ECM synthesis and degradation. Importantly, biomarker measurements alone are insufficient. Assessments of both diastolic and systolic function should be incorporated to determine whether treatment improves cardiac performance and compliance rather than merely reducing collagen deposition. Where feasible, noninvasive cardiac imaging and circulating fibrosis biomarkers may serve as complementary measures for longitudinal assessment of disease progression and therapeutic response.

Most current evidence is derived from rodent models and immortalized cell lines, which are limited by species-specific differences and cannot fully reproduce the pathological features. To narrow this translational gap, future validation studies should prioritize human-relevant experimental platforms, including primary human cardiac fibroblasts, human induced pluripotent stem cell-derived cardiomyocytes and engineered cardiac tissues, as well as precision-cut human cardiac slice models. These human-derived systems enable more accurate evaluation of compound efficacy, target specificity and cellular toxicity, thereby facilitating the identification of credible natural product-derived antifibrotic candidates and reducing false-positive findings in preclinical research.

## 6. Conclusions and Future Perspectives

This review synthesizes recent advances in the therapeutic potential of natural products against MF. In contrast to conventional anti-remodeling agents that predominantly target hemodynamic improvement or neurohumoral suppression, bioactive natural products, encompassing alkaloids, flavonoids, terpenoids, saponins, phenolic acids, and polysaccharides, exhibit inherent multi-component, multi-target, and multi-pathway characteristics. The prevailing mechanisms elucidated herein involve attenuating cardiac fibroblast activation, inhibiting ECM deposition, mitigating oxidative stress and inflammation, restoring mitochondrial homeostasis, modulating EndMT, and regulating autophagic flux. Critically, these mechanisms are not isolated; they are functionally interwoven through core signaling axes—particularly the TGF-β/Smad cascade—wherein inflammatory cytokines, ROS accumulation, mitochondrial dysfunction, RAAS activation, EndMT, and ECM remodeling form a self-perpetuating pathological network driving MF progression.

MF is a dynamic and context-dependent pathology, embodying both adaptive and maladaptive roles. While reparative fibrosis preserves ventricular integrity in the acute post-injury phase, its persistence under chronic pressure overload, metabolic stress, or ischemia becomes unequivocally maladaptive. Consequently, intervention timing is paramount. Early inflammatory responses may facilitate necessary scar formation, whereas sustained macrophage–fibroblast crosstalk and chronic activation of NF-κB/NLRP3 and TGF-β/Smad signaling drive myofibroblast persistence and pathological ECM accrual. Similarly, autophagy and mitophagy are contextually dichotomous: initially cytoprotective by eliminating damaged organelles, yet potentially deleterious when dysregulated. Future investigations must therefore prioritize stage-specific and cell-type-targeted interventions aligned with disease kinetics.

It is noteworthy that this review deliberately focused on peer-reviewed pharmacological and mechanistic evidence; consequently, the patent literature was not systematically evaluated. While patents offer insights into novel entities and formulations, the variability in experimental rigor and the often preliminary nature of intellectual property claims render them unsuitable for the mechanistic synthesis intended here. A comprehensive patent-landscape analysis is reserved for future investigation to complement these scientific findings.

In summary, natural products represent a valuable reservoir of antifibrotic lead compounds and potential adjunctive therapies for MF. The field must now transition from descriptive compound cataloguing toward an integrated evidence framework. This framework should synergize ethnomedical context, stringent source authentication, detailed phytochemical characterization, mechanistic validation, safety pharmacology, and human-relevant modeling. Such a paradigm shift will not only deepen our understanding of MF pathobiology but also accelerate the development of natural product-derived interventions with defined mechanisms and high translational fidelity.

## Figures and Tables

**Table 1:** Evidence map of representative natural products with myocardial-fibrosis relevance.

Class and Candidate	Reported Source Context	Model/Fibrosis Evidence	Principal Mechanism	Evidence Interpretation and Key Citation
Alkaloid: Berberine	*Coptis chinensis*/*Phellodendron amurense*	Doxorubicin-induced myocardial injury and fibrosis; collagen and oxidative-stress endpoints	Nrf2 activation; mitochondrial protection; oxidative-stress reduction	Direct preclinical; exposure and human relevance remain uncertain [23]
Alkaloid: Tetramethylpyrazine	*Ligusticum chuanxiong*-derived compound	Post-myocardial infarction ventricular remodeling	SIRT1/p300/YY1/sST2 axis; immune microenvironment modulation	Direct preclinical [24]
Alkaloid: Lycorine	*Lycoris*/*Narcissus* spp.	Ang II-induced cardiac remodeling and inflammation	PI3K/Akt/NF-κB suppression	Direct preclinical; ethnocardiovascular rationale is weaker than for classic cardiovascular herbs [25]
Alkaloid: Leonurine	*Leonurus japonicus*-derived compound	Cardiac fibrosis model with cardiomyocyte pyroptosis	TGF-β/Smad2 modulation; pyroptosis inhibition	Direct preclinical; mechanism should be supported by causal blocking experiments [26]
Alkaloid: Palmatine	*Coptis*/*Phellodendron*-derived compound	Cardiac fibroblast activation and fibrosis	STAT3 inhibition	Direct preclinical; needs PK and safety contextualization [27]
Polysaccharide: GPS-1-1	*Glycyrrhiza uralensis* polysaccharide	MF model; ECM deposition and fibroblast migration endpoints	MAPK/PI3K/Akt modulation	Direct preclinical; structural characterization is essential [28]
Polysaccharide: SRP-1	Stellariae Radix polysaccharide	MF model; Col I/III and α-SMA endpoints	TGF-β/Smad suppression	Direct preclinical; botanical identity and polysaccharide structure should be verified [29]
Polysaccharide: Morchella polysaccharide	*Morchella esculenta*-derived polysaccharide	Post-myocardial infarction inflammation and fibrotic repair	Gut microbiota-derived 12-HEPE axis	Direct preclinical; microbiota-mediated mechanism requires replication [30]
Polysaccharide: Lentinan	*Lentinus edodes*-derived β-glucan	Ang II-induced myocardial remodeling	LMP7/SOCS3 axis; STAT3, ERK, Akt, NF-κB and TGF-β signaling	Direct preclinical; batch consistency and immune effects are relevant [31]
Flavonoid: Formononetin	*Astragalus*/*Pueraria*-associated isoflavone	Isoproterenol-induced MF	Mitochondrial dysfunction improvement	Direct preclinical; source-specific relevance should be clarified [32]
Flavonoid: Calycosin	*Astragalus*-derived isoflavone	Post-myocardial infarction fibrosis and cardiac dysfunction	TGFBR1 signaling suppression; reduced fibroblast proliferation and collagen deposition	Direct preclinical; strong mechanistic fit with fibroblast biology [33]
Flavonoid: Liquiritin	Licorice-derived flavonoid glycoside	Post-myocardial infarction cardiac fibrosis	CCL5/NF-κB suppression	Direct preclinical; inflammatory mechanism requires cell-specific validation [34]
Flavonoid: Apigenin	Medicinal and dietary plant flavone	Isoproterenol-induced MF	miR-122-5p/miR-155-5p and NF-κB/TGF-β1/Smad modulation	Direct preclinical; dietary exposure may not match experimental doses [35]
Flavonoid: Puerarin	*Pueraria lobata*-derived isoflavone	Isoproterenol- and pressure-overload-associated fibrosis models	TGF-β1 suppression; Nrf2 activation; EndMT modulation	Direct preclinical; relatively consistent cardiovascular rationale [36,37,38]
Phenolic/polyphenol: Curcumin	*Curcuma*-derived polyphenol	Cardiac fibroblast and macrophage–fibroblast crosstalk models	Smad2/p38; IL-18/TGF-β1/p-Smad2/3; EndMT-related Nrf2 axis	Cell-based/supportive; low bioavailability is a major translational barrier [39,40]
Phenolic acid: Salvianolic acid B	*Salvia miltiorrhiza*-derived phenolic acid	Diabetic cardiomyopathy and fibroblast activation	Smad7 stabilization and TGF-β1 blockade	Direct/supportive preclinical; PK and formulation issues require attention [41]
Saponin: Panaxatriol saponin	*Panax notoginseng*/*Panax ginseng* saponin fraction	Myocardial infarction-induced cardiac fibrosis	Keap1/Nrf2 targeting; reduced oxidative stress and fibroblast activation	Direct preclinical; fraction composition should be standardized [42]
Saponin: Chikusetsu saponin IVa	*Panax japonicus* saponin	Isoproterenol-induced MF	AMPK/mTOR/ULK1-mediated autophagy activation	Direct preclinical; autophagic flux evidence should be checked [43]
Saponin mixture: tea saponins (oleanane-type pentacyclic triterpenoid glycosides)	Purified *Camellia oleifera* seed-meal saponin fraction	Hypertension-induced MF	ACE/AT1R/TGF-β/NF-κB axis suppression	Direct preclinical; a chemically heterogeneous mixture rather than a single compound; composition requires standardization [44]
Terpenoid: Celastrol	*Tripterygium wilfordii*-derived triterpenoid	Myocardial infarction-induced fibrosis	NLRP3 inflammasome inhibition	Direct preclinical; toxicity window is a central limitation [45]
Sesquiterpenoid: Atractylenolide III	*Atractylodes macrocephala*-derived lactone	Post-myocardial infarction fibrosis	RhoA/ROCK1 and ERK1/2 suppression	Direct preclinical [46]
Diterpene glycoside: Stevioside	*Stevia rebaudiana*-derived glycoside	Isoproterenol-induced MF	NF-κB/TGF-β1/Smad suppression	Direct preclinical; cardiometabolic exposure and dosing need clarification [47]
Triterpenoid: Corosolic acid	*Eriobotrya japonica* leaf-associated triterpenoid	Post-myocardial infarction cardiac fibrosis and dysfunction	AMPKα-associated inflammation and oxidative-stress regulation	Direct preclinical [48]

## Data Availability

No new data were created or analyzed in this study. Data sharing is not applicable to this article.

## Abbreviations

12-HEPE	12-hydroxyeicosapentaenoic acid
ACE	angiotensin-converting enzyme
ACTA2	actin alpha 2, smooth muscle
Akt	protein kinase B
AMPK	AMP-activated protein kinase
Ang II	angiotensin II
AP-1	activator protein 1
AT1R	angiotensin II type 1 receptor
AUC	area under the concentration–time curve
CCL5	C-C motif chemokine ligand 5
CD31	cluster of differentiation 31
CHOP	C/EBP homologous protein
COL1A1	collagen type I alpha 1 chain
COL3A1	collagen type III alpha 1 chain
CTGF	connective tissue growth factor
CYP	cytochrome P450
DNA	deoxyribonucleic acid
ECM	extracellular matrix
EGCG	epigallocatechin-3-gallate
EndMT	endothelial-to-mesenchymal transition
eNOS	endothelial nitric oxide synthase
ERK1/2	extracellular signal-regulated kinase 1/2
FOXO3	forkhead box O3
FSP1	fibroblast-specific protein 1
GDMT	guideline-directed medical therapy
GLP-1R	glucagon-like peptide-1 receptor
GR	glucocorticoid receptor
GRP78	glucose-regulated protein 78
GSH	glutathione
GSH-Px	glutathione peroxidase
GTPBP4	GTP-binding protein 4
HO-1	heme oxygenase 1
IGFBP2	insulin-like growth factor-binding protein 2
IL	interleukin
JAK	Janus kinase
JNK	c-Jun N-terminal kinase
Keap1	Kelch-like ECH-associated protein 1
LC3	microtubule-associated protein 1 light chain 3
LMP7	low-molecular-mass polypeptide 7/immunoproteasome subunit beta5i
MAPK	mitogen-activated protein kinase
MDA	malondialdehyde
MF	myocardial fibrosis
miR	microRNA
MitoQ	mitochondria-targeted coenzyme Q antioxidant
MMPs	matrix metalloproteinases
MTA3	metastasis-associated protein 3
mTOR	mechanistic target of rapamycin
NADPH	nicotinamide adenine dinucleotide phosphate
NF-κB	nuclear factor-κB
NLRP3	nucleotide-binding oligomerization domain-like receptor family pyrin domain-containing 3
NOX4	NADPH oxidase 4
Nrf2	nuclear factor erythroid 2-related factor 2
NRG-1	neuregulin-1
PD	pharmacodynamics
PK	pharmacokinetics
p300	E1A binding protein p300
p62	sequestosome 1
PI3K	phosphatidylinositol 3-kinase
PINK1	PTEN-induced kinase 1
PPARγ	peroxisome proliferator-activated receptor gamma
RAAS	renin–angiotensin–aldosterone system
RhoA	Ras homolog family member A
ROCK	Rho-associated coiled-coil-containing protein kinase
RNA-seq	RNA sequencing
ROS	reactive oxygen species
SIRT	sirtuin
SIRT1	sirtuin 1
SIRT3	sirtuin 3
Smad	suppressor of mothers against decapentaplegic
SOCS3	suppressor of cytokine signaling 3
SOD	superoxide dismutase
SRP-1	Stellariae Radix polysaccharide 1
STAT3	signal transducer and activator of transcription 3
STZ	streptozotocin
sST2	soluble suppression of tumorigenicity 2
TAC	transverse aortic constriction
TGF-β	transforming growth factor-β
TGFBR1	transforming growth factor-β receptor 1
TIMPs	tissue inhibitors of metalloproteinases
TIMP-2	tissue inhibitor of metalloproteinases 2
TNF-α	tumor necrosis factor alpha
ULK1	unc-51-like autophagy activating kinase 1
VE-cadherin	vascular endothelial cadherin
Vps34	vacuolar protein sorting 34
Wnt	wingless/integrated
YY1	Yin Yang 1
α-SMA	α-smooth muscle actin
